# Spatiotemporal Overlap between the European Brown Hare and Its Potential Predators and Competitors

**DOI:** 10.3390/ani11020562

**Published:** 2021-02-21

**Authors:** Andrea Viviano, Emiliano Mori, Niccolò Fattorini, Giuseppe Mazza, Lorenzo Lazzeri, Alessandra Panichi, Luigi Strianese, Walid Fathy Mohamed

**Affiliations:** 1 Dipartimento di Scienze Agrarie, Università degli Studi di Pisa, 56124 Pisa, Italy; a.viviano@studenti.unipi.it; 2 Consiglio Nazionale delle Ricerche, Istituto di Ricerca sugli Ecosistemi Terrestri, Via Madonna del Piano 10, 50019 Sesto Fiorentino, Italy; 3 Dipartimento di Scienze Ambientali e Politiche–ESP, Università di Milano, 20133 Milano, Italy; niccolo.fattorini@gmail.com; 4 CREA Research Centre for Plant Protection and Certification, Cascine del Riccio, 50125 Firenze, Italy; giuseppe.mazza@crea.gov.it; 5 Dipartimento di Scienze della Vita, Università di Siena, 53100 Siena, Italy; lazzerilorenzo12@gmail.com; 6 Dipartimento di Biologia, dell’Università di Firenze, 50019 Sesto Fiorentino, Italy; alessandra.panichi@stud.unifi.it; 7 Associazione Nazionale Libera Caccia, 58100 Grosseto, Italy; info@elettronicastrianese.com; 8 Department of Biological and Geological Sciences, Faculty of Education, Ain Shams University, Roxy, Cairo 11566, Egypt; walidfathy2021@gmail.com

**Keywords:** activity rhythms, camera-traps, *Lepus europaeus*, moonlight avoidance, niche partitioning, spatiotemporal behavior, predator–prey systems

## Abstract

**Simple Summary:**

Predator-prey relationships and competition shape interspecific coexistence in wildlife communities. So far, most published studies have focused on large carnivores and their prey, whereas little is known about medium and small-sized mammal communities. The European brown hare *Lepus europaeus* is a widespread species in Europe and is part of the diet of many birds of prey and mammalian carnivores of all sizes. Furthermore, competition with other herbivorous mammals at feeding sites has also been suggested. In an area in Central Italy, we have assessed spatiotemporal overlap among brown hare and its potential predators (red fox *Vulpes vulpes,* pine marten *Martes martes*, domestic cat *Felis catus,* and domestic dog *Canis familiaris*) and a competitor (roe deer *Capreolus capreolus*). We showed that, outside a fenced area excluding predators and competitors, brown hares become more nocturnal and more active on dark nights to limit encounters with predators, and that they adopt spatial partitioning to avoid competitors, as expected by ecological theory.

**Abstract:**

Analysis of spatiotemporal partitioning is pivotal to shed light on interspecific coexistence. Most research effort has involved large-sized carnivores and their prey, whereas little attention has been devoted to lagomorphs. We assessed spatiotemporal overlap among the European brown hare *Lepus europaeus* and its potential competitors and predators through camera-trapping in an area in Central Italy. We estimated the interspecific patterns of the spatiotemporal activity rhythms of brown hares, its potential predators (the red fox *Vulpes vulpes,* the pine marten *Martes martes*, the domestic cat *Felis catus,* and the domestic dog *Canis familiaris*), and a competitor, the roe deer *Capreolus capreolus*. Brown hare activity was studied in natural conditions as well as in a fenced area that excluded terrestrial predators and competitors. Free-ranging hares developed a more nocturnal behavior to avoid diurnal predators (i.e., domestic carnivores and martens). Although high temporal overlap was observed between free-ranging brown hares and both red foxes (82%) and roe deer (81%), hares avoided fox by being more active on darkest nights, as well as avoided roe deer through spatial partitioning. We suggest that hares may adapt their spatiotemporal behavior to avoid potential predators and competitors.

## 1. Introduction

Interspecific interactions and niche partitioning are known to shape animal communities, with predators adopting behavioral strategies to maximize the probability of killing their prey, and with prey developing antipredator tactics to limit the risk of being killed [1,2]. Similarly, interspecific competition may trigger behavioral plasticity, forcing competitors to adapt their behaviors to avoid interactions and to fulfill their life-history requirements [3].

The use of camera-traps has provided important ecological information concerning the spatiotemporal behavior of wild species, including species of conservation interest [4], rare/elusive species [5,6,7], as well as problematic species requiring management action such as alien species [8,9]. It has been shown that 30–100 independent camera-trap records per species for each season or year may be sufficient to estimate activity rhythms of wildlife, with results comparable to those obtained from GPS or radio-tracked animals [10]. Camera-traps have become cost-effective tools and may provide researchers with a good amount of data [11]. In the last ten years, many studies dealing with the activity rhythms of mammalian species and their interspecific overlap have been published [12,13,14,15,16]. These studies have mostly focused on prey-predator relationships involving large carnivores [16,17,18], intraguild interactions [19,20,21,22,23], population density and structure [24], and activity bouts at artificial feeding sites [25,26]. Conversely, little research has investigated spatiotemporal interactions amongst mesocarnivores and small-sized mammals, and between them and their potential predators [27,28].

The European brown hare *Lepus europaeus* is a small mammal species widely distributed throughout Eurasia [29], where it represents an important prey species for many vertebrates, including carnivores and occasionally rodents amongst mammals, as well as corvids and raptors amongst birds [30,31,32]. This species is typical of agroecosystems, characterized by traditional agricultural practices, mostly used for feeding, alternated to scrublands, hedgerows, and woodlands where shelter sites occur [33]. The European brown hare has been introduced worldwide for hunting purposes, particularly during the last century; currently, introduced populations occur in America and Oceania [34]. Despite this widespread introduction effort, brown hare populations have been declining in several parts of the European range, possibly because of environmental pollution, parasite-mediated competition with introduced species, and poaching [35,36,37]. Given the economic interests of this lagomorph as a popular game species, hunting agencies and associations have promoted studies on the habitat requirements of the brown hare, as well as programs of environmental improvement to keep the species at high densities [38,39,40]. Habitat structure and crop variety have been reported as important factors promoting local hare abundance [38,39]. Environmental improvement programs increasing habitat heterogeneity have succeeded in augmenting the local hare populations [39,40,41,42], even without predator culling [43]. Prey species may avoid predators by increasing their activity on the darkest nights, whereas carnivores may be more successful in hunting on bright moonlight nights [7,12,19]. A high synchronization has been observed in Southern Italy between the hare and the grey wolf *Canis lupus* [16]. Accordingly, in Turkey, activity rhythms of the Eurasian lynx *Lynx lynx* were temporally synchronized with those of the brown hare, supporting the brown hare as the main local prey species of the Eurasian lynx [44], although the authors did not test the effect of moon phases. Additionally, the presence of small carnivores, potential hare predators, has been suggested to alter the spatial behavior of this lagomorph, which shifts its spatial behavior to open areas with short vegetation where detection of potential predators is highest [45,46], or in areas rarely used by predators [47]. The European brown hare is reported to be mainly nocturnal, with activity peaking mostly in the first part of the night or in the crepuscular hours [48,49,50,51]. However, no information is available on its temporal adaptations to limit encounters with its main predators, i.e., mesocarnivores [30,52,53,54,55]. In this study, we aimed at assessing the spatiotemporal mechanisms of interspecific coexistence between the brown hare and co-occurring medium-sized mammals, including four mesocarnivores reported as potential predators of hares [30] and one potential competitor species, the roe deer *Capreolus capreolus* [56]. We predicted that (i) a high spatiotemporal overlap would occur between predators and hares; (ii) that hares would avoid roe deer either spatially or temporally; and that (iii) interspecific temporal overlap will be altered by the moon’s phases, with predators being mostly active on bright nights, and prey being most active on the darkest ones.

## 2. Materials and Methods

### 2.1. Study Area

**Figure 1:**
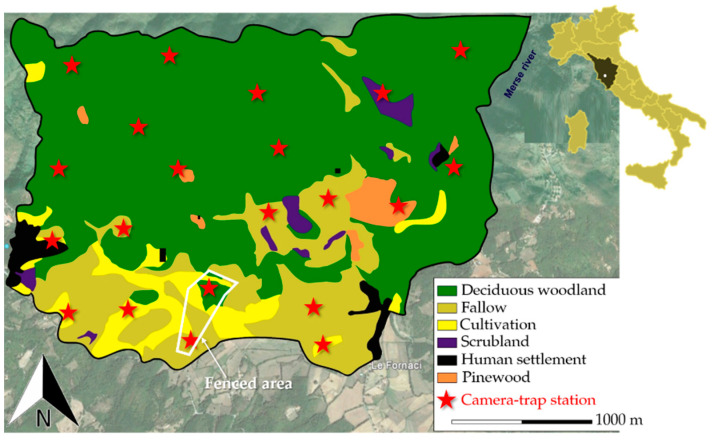
Location of the study area, habitat type composition, and position of camera-trap stations.

We conducted our field-work in 2016 and 2017, in a special conservation area of Southern Tuscany, Central Italy (Poggi di Prata: 43.083° N, 10.989° E; 1350 ha, 475–903 m above sea level: Figure 1). In our survey period, the mean annual rainfall was 670 ± 26 mm and the mean annual temperature was 15.9 ± 7.7 °C. About 65% of the study area was composed of deciduous woodlands (*Quercus cerris* L., *Castanea sativa* Mill., *Ostrya carpinifolia* Scop., and *Carpinus betulus* L.). Scrubland (*Juniperus communis* L., *Rubus* spp., and *Spartium junceum* L.: about 2%) created belts around woodlands. Open habitats, i.e., fallows and cultivations (mostly sunflowers and cereals), represented respectively 19% and 8% of the study area. Pinewood (*Pinus nigra* J.F. Arnold and *Cupressus arizonica* E. Greene) and human settlements occurred in the remaining part of the study area. A five-hectare area surrounded by a 2.5 m high, fine-mesh (5 cm) fence [57,58], used for small game species acclimatization before restocking (European brown hare, common pheasant *Phasianus colchicus,* and red-legged partridge *Alectoris rufa*), was present in the southern part of the study site. The uppermost part of the fence was further guarded with a 30 cm section of net projecting at right angles to the outside of the fence. Thus, the fenced area excluded terrestrial carnivores, although it was still accessible to raptor birds (e.g., buzzards *Buteo buteo* and hen harriers *Circus cyaneus*). Previous intensive camera-trapping in this area provided evidence for the absence of mesopredators and large-sized mammals [57,58]. About 25–30 hares/year are kept in the enclosure for 2–3 months. Hunting guards report that every year nearly 20% of these hares are preyed on by buzzards in the fenced area. Several individuals of brown hare remain in the fenced area throughout the year, and camera-trapping in the fenced area provided us with a control area. The study area hosts a rich community of vertebrate species, with over 30 mammal species [59], including the European brown hare. Potential terrestrial predators of the brown hare in our study area are the red fox*,* the pine marten, and a few free-ranging domestic dogs and cats. Data on the local population densities of these species are not available. Previous data on the local diet of the red fox confirmed that the brown hare is a potential prey of this canid [60]. Similarly, several brown hares have been killed locally by domestic carnivores, whereas this lagomorph was not detected in the local diet of the wildcat *Felis silvestris* [61].

### 2.2. Camera-Trap Sampling Design

Camera-traps (*N* = 4, Multipir 12 scouting camera) were placed at 20 sampling stations (Figure 1) on trees or rocks, depending on the area, and tied with ropes and chains. Stations were separated from one another by at least 700 m, to limit pseudoreplication bias [19]. Camera-traps were checked once every 10 days for 2 years (2016–2017), to download data from SDs and refresh batteries. Camera-traps were kept at each station for 10–12 days and then randomly allocated around the 20 sites. In other words, each station was equipped with camera-traps for each of the four seasons, to ensure temporally-balanced sampling [16]. Camera-traps were put on the closest path/track to predetermined random points selected within a regular grid [16], covering the whole study area, at a height of ~80 cm from ground level, and were kept active round-the-clock, to take one video of 60 s/event. All camera-traps were hidden with vegetation and tree bark to limit neophobic reactions. As an experimental control, two more camera-traps were also kept active 24 h/day in the fenced area throughout the study period. 

### 2.3. Analysis of Spatiotemporal Overlap

Analyses were carried out on both annual and seasonal (cold vs warm period) scales. We identified a cold period (October-March: mean temperature, 9.0 ± 3.0 °C) and a warm one (April–September: mean temperature, 17.0 ± 5.0 °C). We defined activity as the cumulate period that animals spend outside resting sites, regardless of their behavior [9,62]. For all videos, we recorded the date and the solar hour of capture. The use of the solar hour allows a better evaluation of activity patterns than the legal hour, as it is only defined by the position of the sun in the sky. We considered as an “independent event” all videos of the same species from the same camera-trap station recorded in ≤30 min, to limit pseudoreplication bias [3,16,23]. When more than one video of the same species was recorded by the same camera-trap in ≤30 min, we retained only the video placed in the median-time between the first and the last video. All the other records of the same species occurring in ≤30 min per site were thus deleted from the final dataset to avoid pseudo-replications [62]. We used the package overlap [62] in R 3.5.1 [63] and calculated the interspecific temporal overlap by estimating the coefficient of overlap (Δ = 0, no overlap, Δ = 1, total overlap [62]) between temporal activity patterns of all pairwise combinations of species. Two Δ estimators can be used, calculated with different algorithms depending on sample size. In detail, we computed the Δ_4_ estimator when both samples of the pairwise comparison included ≥75 independent records; we used the Δ_1_ estimator when records were <75 for at least one species of the pair [62]. Temporal overlaps of activity patterns were ranked by considering a high overlap with Δ > 0.75, a moderate overlap if 0.50 < Δ < 0.75, and a low overlap if Δ < 0.50 [23]. The 95% confidence interval (hereafter, 95% CI) of the coefficient estimator was calculated with 10,000 bootstrap replicates of activity patterns [17]. We performed a Hermans-Rasson test to evaluate whether a random activity pattern was exhibited over the 24 h [17,64,65]. For each pairwise comparison between species, an additional bootstrap analysis was performed to estimate the probability that two sets of circular observations belonged to the same distribution, using the package activity [17] in R 3.5.1. Moonlight level was recorded to assess whether it affected the temporal activity patterns of studied species. Moon phases were classified as follows: phase (1) from new moon to ¼; phase (2) from ¼ to ½; phase (3) from ½ to ¾; and phase (4) over ¾ and full moon. Then, we performed a chi-squared test on the number of detections recorded during each moon phase, to assess whether they were uniform throughout the four moonlight levels [19]. Interspecific spatial overlap was estimated through the Pianka index (O = 0, no overlap; O = 1, total overlap: [66]), considering the proportion of records of each species at different camera-trapping stations [19]. Each camera-trap station was used as a sampling unit to test spatial overlap among species following previous studies [16,19].

This index was computed through the formula:

O*_jk_* = (Σp*_ij_* × p*_ik_*)/(Σp*_ij_*^2^ × Σp*_ik_*^2^)^1/2^

where p*_ij_* is the proportion of records of species *j* and p*_ik_* is the proportion of records of the species *k*.

## 3. Results

**Figure 2:**
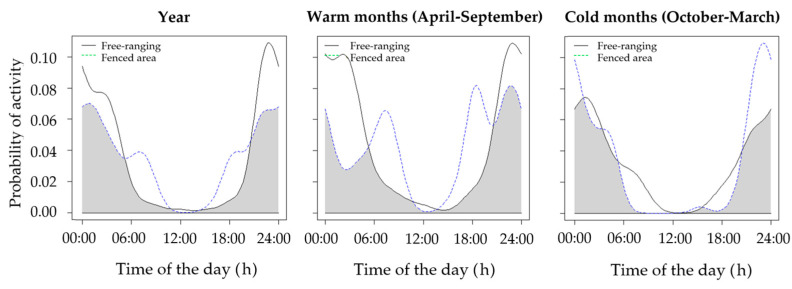
Overlap of activity rhythms of the brown hare in natural conditions and in the fenced area.

Our survey included a total of 2824 camera-trap days (i.e., *N* of camera-traps × *N* days they were active). We obtained 266 records of brown hare at 9 camera-trap stations, 129 in the fenced area (*N* = 44 in the warm period, *N* = 85 in the cold period) and 137 outside the fenced area (*N* = 69 in the warm period, *N* = 68 in the cold period). The brown hare was mainly active in dark hours in both fenced and non-fenced areas, with a few bouts of diurnal activity, mostly in the fenced area, in the warm months (Figure 2). Throughout the year, activity patterns were significantly different from a random pattern of activity (Hermans-Rasson tests; *r* = 73.22–79.42, *p* < 0.001), peaking in the first part of the night (23:00–00:00) in both the fenced and the non-fenced areas. The temporal overlap of activity patterns between fenced and non-fenced areas was very high in all seasons (Table 1), although peaks of activity at dawn and dusk were observed only in the fenced area. Annual activity of hares outside the fenced area was compared with that of red fox (*N* = 127 records), pine marten (*N* = 59 records), domestic dog (*N* = 88 records), domestic cat (*N* = 35 records), and roe deer (*N* = 1531 records).

**Figure 3:**
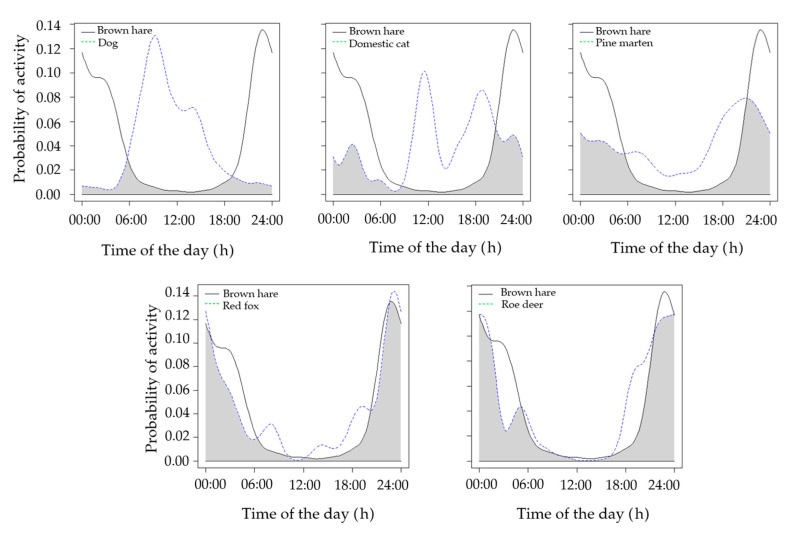
Annual overlap of activity rhythms between the brown hare and its potential terrestrial predators and competitors.

The annual activity of hares outside the fenced area was compared with that of the red fox (*N* = 127 records), pine marten (*N* = 59 records), domestic dog (*N* = 88 records), domestic cat (*N* = 35 records), and roe deer (*N* = 1531 records). We detected a high overlap with the red fox and the roe deer, an intermediate overlap with the pine marten, and a low overlap with domestic carnivores (Table 1; Figure 3). 

Brown hares avoided bright nights throughout the year and were mostly active in phase 2 in natural conditions (χ^2^ = 59.35, dof = 3, *p* < 0.001); conversely, hare activity did not depend on moon phase in the fenced area (χ^2^ = 6.92, dof = 3, *p* > 0.05). The red fox was mostly active on bright nights (χ^2^ = 66.48, dof = 3, *p* < 0.001). The patterns of activity rhythms of the other terrestrial predators and of the roe deer were not related to moon phase (domestic dog, χ^2^ = 2.16, dof = 3, *p* > 0.25; pine marten, χ^2^ = 3.53, dof = 3, *p* > 0.25; roe deer, χ^2^ = 0.51, dof = 3, *p* = 0.08). Indices of spatial overlap between the hare and other species were always lower than 0.50, with the exception of the domestic dog (O*_jk_* = 0.77; Table 2).

## 4. Discussion

Our findings provide evidence that European brown hares can shift their temporal activity patterns when terrestrial predators are present. However, our study has limitations due to our relatively small set of camera-traps, requiring confirmation by a larger sample of camera-traps over a larger area. On the other hand, given the presence of several ditches around cultivated patches within the study area, which prevent movements of hares between patches, different individuals should have been recorded at different stations [19]. Furthermore, different habitat types may host different predators [14,15,16], with domestic carnivores most often recorded close to human settlements and country houses. In our study, we could not test for potential differences in activity rhythms amongst different habitat types due to the low sample size, which needs to be investigated by future research. Accordingly, where hare predators are limited to birds of prey [57,58,67], i.e., in the fenced area, some crepuscular activity is observed, particularly at birth peak, in the warm months, when surveillance behavior needs to be increased [68,69]. We cannot rule out that this behavior may differ from that observed in natural conditions, because hares in the fenced area came from breeding cages and were previously fed by humans in daylight hours. However, this shift to crepuscular activity could be adopted by hares where predation pressure is low, as it is similar to that observed where predator culling programs occur [50,51]. In the fenced area, no terrestrial predator has been camera-trapped during the two-year survey, confirming that they were not locally present [57,58]. We conducted a more intensive sampling in the fenced area to confirm this result. This sampling bias may have partially altered the assessment of activity rhythms with respect to natural sites (e.g., through a reduction in variance), but differences between fenced and non-fenced areas are too remarkable to be due only to a different sampling protocol.

In general, hares were confirmed to be mostly nocturnal, in line with previous studies [44,48,49,50,51]. Mammalian predators and competitors may however play a major role in structuring the spatiotemporal behavior of this important prey species. Spatial niche partitioning and temporal segregation may allow coexistence between mesocarnivore and medium-sized species [3,15,19,21,23], particularly for those species showing high dietary overlap [19,21]. Despite being reported as a species also occasionally killed by free-ranging domestic cats, even in our study area [70], a low spatiotemporal overlap was observed between these species and the brown hare. Hares may be captured by diurnal domestic carnivores also at shelter sites, i.e., while resting, as confirmed by leverets commonly killed by domestic cats. Conversely, a high spatial overlap was observed with domestic dogs which were mostly represented by shepherd dogs and hounds used for wild boar and hare hunting. Similarly, the pine marten showed temporal and spatial partitioning with the brown hare, as representing a woodland-dwelling, mainly diurnal carnivore in our study area [19]. A high spatiotemporal overlap was observed between the brown hare and the red fox. In Central Italy, the red fox is mostly a fruit-consumer [60,71,72], although in some areas hares may play an important role in its diet, particularly where other food items are scarce [60,73]. Furthermore, foxes are perceived by brown hare as a threat, whatever the predation rate on this species [47]. Thus, outside the fenced area, the brown hare tends to use similar areas to those used by foxes, and both species are mostly nocturnal. Conversely, in areas with a rich community of rodents and lagomorphs, red foxes and hares show a low temporal overlap [28]. Accordingly, in these areas, lagomorphs do not avoid bright moonlight nights [28]. In general, predators tend to be more active on bright moonlight nights, when hunting success is the highest [18,19,74,75]. Conversely, prey species tend to avoid predation through several survival tactics involving spatiotemporal partitioning [70,71]. For instance, lagomorphs and rodents are reported to limit the risk of being preyed upon by concentrating their movements on the darkest nights and/or in sheltered habitats [76,77,78,79]. In our study system, outside the fenced area, hares tend to avoid foxes mostly by being active on the darkest nights, when the effectiveness of fox predation is reported to be lowest and when foxes are the least active [80]. We suggest that this should be seen as an adaptation to thrive in habitats where predation/competition risk is high, as no moonlight avoidance by brown hares was observed in the fenced area.

Interspecific competition occurs when two or more species share scarce resources resulting in negative effects on physiology, growth, and survival of at least one of them [80,81]. Dietary overlap has been reported between ungulates and lagomorphs [54,81,82,83,84,85], with the latter often forced by larger herbivores to use suboptimal areas [84]. The roe deer was the only cervid occurring in our study area, apart from the rare fallow deer *Dama dama* [59]. Competition between roe deer and the brown hare has been suggested to occur mostly for feeding resources [56]. In natural communities, interspecific competition can be avoided by niche partitioning [80]; in our study area, brown hares and roe deer shared similar temporal patterns of activity, being both crepuscular and nocturnal. However, spatial partitioning was high, with only 16% of spatial overlap, which may reduce interspecific encounters at feeding sites, supporting earlier evidence [56].

## 5. Conclusions

The European brown hare evolved in predator-rich ecosystems, including a rich community of native and introduced mesocarnivores, as well as with several potential competitors, particularly in Europe [84,85]. In Italy, where our research was conducted, the hare is reported as a prey species for a large number of small and medium-sized carnivores including at least eight mustelids, four canids, and two felid species [30,86], as well as a potential competitor of several species of ungulates and lagomorphs [87]. The high predation pressure and possible interspecific competition to which the brown hare has been subjected during its life-history has likely constrained this species to develop plastic behavioral adaptations in order to survive. Such adaptations may thus include spatiotemporal shifts towards areas and/or temporal niches where predator or competitor avoidance is the highest. European hare populations have been declining across Europe for a long time due to the intensive agriculture and to poaching pressure [88]. Locally, environmental management actions adopted by farmers and hunters have played a key role in the conservation of this lagomorph [89,90], with increasing densities particularly in areas managed by hunting associations and in mountain ecosystems [90,91,92].

## Figures and Tables

**Table 1:** Coefficients and 95% confidence intervals (CIs) of activity overlap between the brown hare and its potential predators and competitors. *p*-values (*p*) obtained by testing whether the two sets of observations come from the same circular distribution are also reported.

Comparison	Overlap Coefficient	95% CI	*p*
Fenced vs. non-fenced brown hare (warm months)	0.64	0.53–0.80	0.02
Fenced vs. non-fenced brown hare (cold months)	0.78	0.68–0.88	0.02
Fenced vs. non-fenced brown hare (whole year)	0.75	0.70–0.87	<0.01
Non-fenced brown hare vs. red fox	0.82	0.74–0.90	<0.01
Non-fenced brown hare vs. domestic cat	0.39	0.29–0.59	<0.01
Non-fenced brown hare vs. domestic dog	0.15	0.13–0.27	<0.01
Non-fenced brown hare vs. pine marten	0.59	0.51–0.75	0.02
Non-fenced brown hare vs. roe deer	0.81	0.77–0.88	<0.01

**Table 2:** Spatial overlap (Pianka index) between the brown hare in natural conditions and its potential predators and competitors.

Comparison	Pianka Index
Brown hare vs roe deer	0.15
Brown hare vs domestic cat	0.36
Brown hare vs domestic dog	0.77
Brown hare vs pine marten	0.18
Brown hare vs red fox	0.46

## Data Availability

Original data are available from the corresponding author upon reasonable request.
